# HC_*n*_H^−^ Anion Chains with *n* ≤ 8 Are Nonlinear and Their Permanent Dipole Makes Them Potential Candidates for Astronomical Observation

**DOI:** 10.3390/molecules27103100

**Published:** 2022-05-12

**Authors:** Ioan Bâldea

**Affiliations:** Theoretical Chemistry, Heidelberg University, Im Neuenheimer Feld 229, D-69120 Heidelberg, Germany; ioan.baldea@pci.uni-heidelberg.de

**Keywords:** astrochemistry, astrophysics, interstellar medium, carbon chains, polyynes, anions, quantum chemistry, radio astronomy, rovibrational spectroscopy, cis and trans isomers

## Abstract

To be detectable in space via radio astronomy, molecules should have a permanent dipole moment. This is the plausible reason why HC_*n*_H chains are underproportionally represented in the interstellar medium in comparison with the isoelectronically equivalent HC_*n*_N chain family, which is the most numerous homologous series astronomically observed so far. In this communication, we present results of quantum chemical calculations for the HC_*n*_H family at several levels of theory: density functional theory (DFT/B3LYP), coupled-cluster expansions (ROCCSD(T)), and G4 composite model. Contradicting previous studies, we report here that linear HC_*n*_H^−^ anion chains with sizes of astrochemical interest are unstable (i.e., not all calculated frequencies are real). Nonlinear cis and trans HC_*n*_H^−^ anion chains turn out to be stable both against molecular vibrations (i.e., all vibrational frequencies are real) and against electron detachment (i.e., positive electroaffinity). The fact that the cis anion conformers possess permanent dipole is the main encouraging message that this study is aiming at conveying to the astrochemical community, as this makes them observable by means of radio astronomy.

## 1. Introduction

Although only representing a small fraction of the extraterrestrial matter, astronomical molecules are very interesting for space sciences because they provide valuable information on the physical and chemical conditions as well as the time evolution of the environments where they are detected. Searching for and confirming the presence of new molecular species plays a role of paramount importance in deepening understanding of astrochemical evolution in the interstellar and circumstellar medium [1].

According to the 2018 census [2], 204 molecules were astronomically detected. Out of them, carbon-based chains represent an important class. With seven members astronomically observed (HCN [3], HC_2_N [4], HC_3_N [5], HC_4_N [6], HC_5_N [7], HC_7_N [8], HC_9_N [9]), the HC_*n*_N chains form the most numerous homologous series detected so far. This is in contrast to the case of the isoelectronically equivalent HC_*n*_H chains [10,11,12,13,14,15,16], out of which only three members (n=2,4,6) were astronomically detected: acetylene H−CC−H [17,18], diacetylene H−CC−CC−H [19], and triacetylene H−CC−CC−CC−H [19].

**Figure 1:**
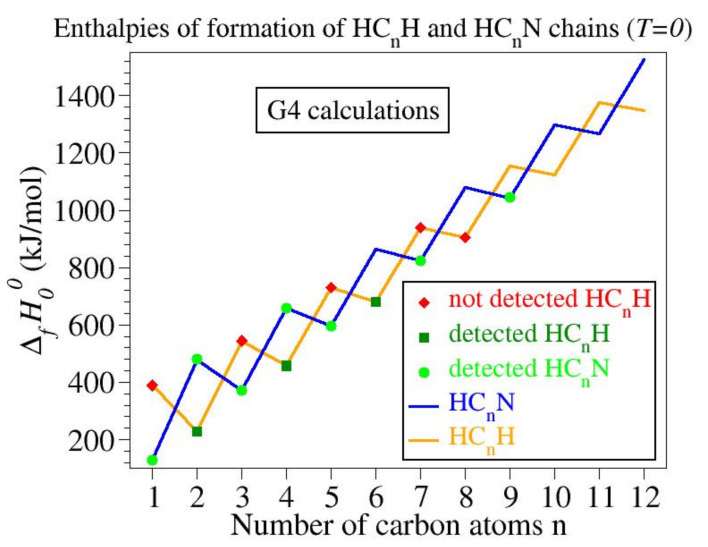
Enthalpies of formation $\Delta_{f}H_{0}^{0}$ of some astronomically observed HC_*n*_N chains are larger than enthalpies of formation of shorterHC_*n*_H chains not yet detected in space. On this basis, there is no reason to assume that HC_*n*_H species are less numerous in space than HC_*n*_N species, although, as visible in this figure, HC_*n*_N molecules already astronomically observed are much more numerous than HC_*n*_H molecules.

However, based on chemical intuition and substantiated below (see discussion related to Figure 1), it would be completely implausible to claim that members of the HC_*n*_N family are more numerous in nonterrestrial environments than members of the HC_*n*_H family. Rather, this underproportional representation of the HC_*n*_H chains found so far in space relative to the HC_*n*_N chains should be related to the complete different difficulty facing HC_*n*_H detection versus HC_*n*_N detection in space.

Possessing permanent dipole, linear HC_*n*_N chains can be detected by radio astronomy, which is *par excellence* the method to observe extraterrestrial molecules that marked the boom in reporting new molecules in space since the early 1960s [2]. According to existing studies—comprising not only neutral HC_*n*_H^0^ [10,20] but also cation HC_*n*_H^+^ [21,22] and anion HC_*n*_H^−^ species [23,24,25]—HC_*n*_H chains are linear. If they are linear (more precisely, centrosymmetric), they have zero dipole moments. Such chains cannot be detected via radio astronomy.

Still, are all HC_*n*_H^−^ chains with molecular sizes of astrochemical interest really linear and centrosymmetric? This was the fundamental question that triggered the investigation whose results will be presented below, and emphasizing anions is part of our recent [26,27,28,29,30] and ongoing effort to understand their role in astrochemistry, which is claimed to even compete with that of the parent neutrals [29,31,32,33].

The prediction of nonlinear HC_*n*_H^−^ anion chains stable against both molecular vibrations (i.e., computed vibrational frequencies are all real) and (excepting n=4) electron detachment (i.e., positive electroaffinity EA>0) and possessing permanent dipole moments (μ≠0) is the main finding reported here. This is the encouraging new message that we aim at conveying to the astrochemical community. To better emphasize it, a series of technical details will be skipped here and deferred to a longer write-up that follows.

## 2. Methods

All quantum chemical calculations in conjunction with this study were done using the GAUSSIAN 16 [34] suite of programs on the bwHPC platform [35].

The enthalpies of formation $\Delta_{f}H_{0}^{0}$ and cis-trans splitting (see Section 3.3) were computed by means of the G4 composite model [36,37]. Recall that in contrast to “simple” models wherein the total electronic energy at frozen geometry—often obtained from optimization at another/lower level of theory (e.g., DFT with smaller basis sets)—is computed by means of a given method (e.g., CCSD(T) and larger basis sets), to achieve high (“chemical”) accuracy, “composite” models (also referred to as compound model chemistries [38]) combine several results obtained via ab initio high-level methods with smaller basis sets with lower-level (DFT) theories using larger basis sets. Within G4, optimization and vibrational frequency calculations are done at the DFT/B3LYP/GTBas3 [34] level. The pertaining zero point energy corrected using an adequate scaling factor as well as thermal corrections to enthalpy and Gibbs free energy are added to the electronic energy estimated at frozen geometry by combining various ab initio methods and basis sets (GAUSSIAN keywords [34] CCSD(T), E4T, FrzG4)/GTBas1, MP4 = FrzG4/GTBas2, MP4 = FrzG4/GTBas3, MP2 = Full/GTLargeXP, HF/GFHFB1, HF/GFHFB2) to obtain values of the total energy, enthalpy, and Gibbs free energy. These estimates turn out to be more accurate than the most elaborate and computationally demanding “simple” ab initio methods (including coupled-cluster (CC) and quadratic configuration interaction (QCI) expansions with singles, doubles and triples corrections (CCSD(T) and QCISD(T), respectively) [36,37].

The values of the adiabatic electron attachment energy EA including corrections due to zero point energy (ZPE) adjusted by means of adequate scaling factors, as standard in compound model chemistries [34,38], were estimated as energies of reaction HC_*n*_H^0^ + e^−^ → HC_*n*_H^−^ at zero temperature, which obviates issues related to the so-called “ion convention” or “electron convention” for the charged species [39,40].

For consistency with previous and ongoing work on related systems [20,27,29,41,42,43,44,45,46] and in order to handle shorter and longer molecules on the same footing, all single-point quantum chemical calculations were carried out at the ROCCSD(T) level of theory, wherein restricted open-shell coupled-cluster expansions include single and double excitations as well as perturbative corrections due to triple excitations [47]. All molecular geometries utilized in these single-point calculations were optimized by means of the three parameter B3LYP hybrid DFT/HF exchange correlation functional [48,49,50,51] and 6-311++G(3df,3pd) basis sets [52,53]; more precisely, restricted RB3LYP for closed shell and unrestricted UB3LYP for open shell species. See Appendix B for further details.

## 3. Results and Discussion

### 3.1. Enthalpies of Formation: HC_*n*_H versus HC_*n*_N

In vein with those noted in the Introduction, let us start by comparing the values of the enthalpies of formation of the HC_*n*_H chains with those of the HC_*n*_N chains. Numerical results obtained using the G4 composite model are collected in Table 1 and depicted in Figure 1. As visible in Figure 1, by and large, neutral HC_*n*_N and HC_*n*_H chains possess comparable enthalpies of formation $\Delta_{f}H_{0}^{0}$. Importantly, some astronomically detected members of the HC_*n*_N family have values of $\Delta_{f}H_{0}^{0}$ larger than values for shorter members of the HC_*n*_H family not yet detected in space. With the grain of salt that formation mechanisms and kinetics are more important for the interstellar synthesis than in laboratory synthesis, the trend seen in Figure 1—corroborated with the important fact that, after all, the HC_*n*_N synthesis requires the presence of extra nitrogen atoms—does by no means substantiate any claim on HC_*n*_N members more numerous in space than HC_*n*_H members. HC_*n*_H’s unfavorable balance in space should not be sought in the production mechanism but rather in the lack of a dipole moment.

### 3.2. Stable HC_*n*_H^−^ Anion Chains with Astrochemically Interesting Sizes Are Nonlinear

Insight gained in conjunction with our recent investigations of astrochemically relevant carbon chain anions [26,27,28,29] made us skeptical that shorter HC_*n*_H^−^ anion chains possess a stable linear geometry, as claimed earlier [23]. Our extensive attempts to optimize HC_*n*_H^−^ anions imposing strict linear conformation confirmed previous results reported for sizes n≥9 [24,25]; we also found that irrespective whether *n* is odd or even, such sufficiently long HC_*n*_H^−^ anions, linear and invariant under spatial inversion, are stable against molecular vibrations, i.e., all calculated frequencies were real.

**Figure 2:**
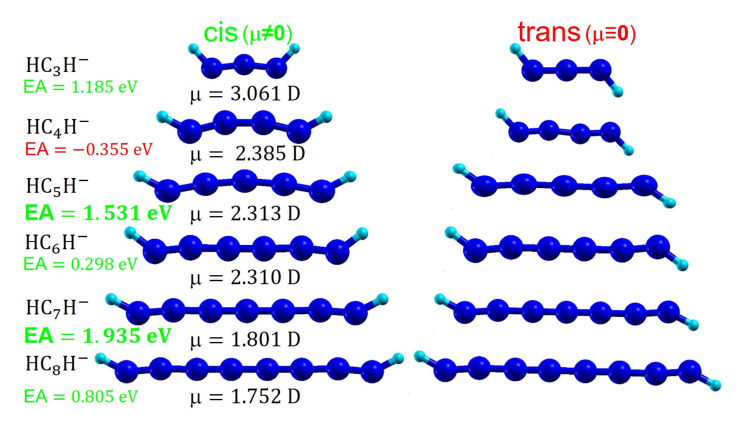
In contrast to the linear conformers, which are unstable against H−C−C bending vibrations at the two molecular ends, cis and trans HC_*n*_H^−^ anion isomers with n≤8 correspond to local energy minima.

However, our calculations disagreed with previous work [23] claiming that HC_4_H^−^, HC_6_H^−^, and HC_8_H^−^ are linear and possess a ${}^{2}\;\Pi_{u}$, ${}^{2}\;\Pi_{g}$, and ${}^{2}\;\Pi_{u}$ ground state, respectively. Whether even (n=4k, n=4k+2) or odd (n=2k+1), we found that strictly linear structures at n≤8 are unstable. Optimization of these anions constrained to be linear invariably ended with molecular conformations having exactly two imaginary frequencies. These two imaginary frequencies correspond to the in-phase and out-of-phase superposition of two vibrational modes, namely the two H−C−C bending modes of the chain ends. In view of this state of affairs, it was not at all surprising to find out that genuine anions’ local energy minima (i.e., all vibrational frequencies real) correspond to cis and trans conformers wherein the two chain ends are bent, as visualized in Figure 2. Full information on the optimized cis and trans anions is presented in Table A2, Table A3, Table A4, Table A5 and Table A6 of Appendix B.

With regard to the specific cases considered in ref. [23], let us mention that at the UB3LYP/6-311++G(3df,3pd) level of theory, we found that the (unstable) linear HC_4_H^−^, HC_6_H^−^, and HC_8_H^−^ conformers lie at 520 meV, 229 meV, and 60 meV above the stable nonlinear conformers. These values are much larger that the cis-trans energy splittings $\Delta_{cis-trans}$ presented in Table 3.

The foregoing analysis made it clear that nonlinear cis and trans anions are “stable” in the sense that they correspond to local energy minima. Equally important for the anions’s “stability” is whether they are also stable against electron detachment, i.e., whether their electroaffinity EA (difference between the total energy of the neutral and the total energy of the anion) is positive. Inspection of Table 2 reveals that with one exception, all computed values of EA computed by us are positive. The exception in question is HC_4_H; this is not surprisingly for small closed-shell molecular species whose anions are rarely stable. Still, given the fact that diacetylene (HC_4_H) was already detected in space [19], HC_4_H^−^’s instability against electron detachment is not so “dramatic” from an astrochemical perspective.

### 3.3. Relevant Properties of Cis and Trans Anions

**Figure 3:**
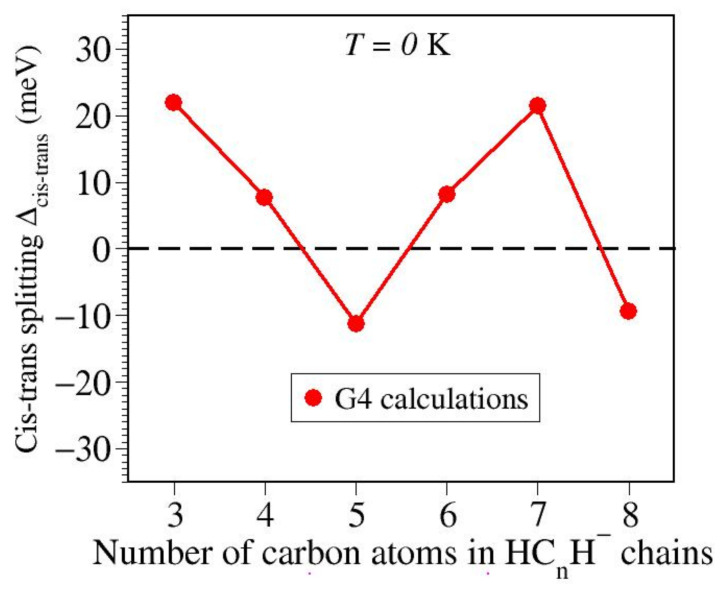
Because the cis-trans energy splitting $\Delta_{cis-trans}\equiv \Delta_{f}H_{0,cis}^{0}\left(\mathrm{HC}nH^{-}\right)-\Delta_{f}H_{0,trans}^{0}\left(\mathrm{HC}nH^{-}\right)$ computed via G4 as enthalpy of isomerization at zero temperature is very small, cis and trans anion conformers are expected to coexist in the interstellar medium.

To obtain the cis-trans energy splitting, we estimated $\Delta_{cis-trans}\equiv \Delta_{f}H_{0,cis}^{0}\left(\mathrm{HC}nH^{-}\right)-\Delta_{f}H_{0,trans}^{0}\left(\mathrm{HC}nH^{-}\right)$ via the G4 composite model. Inspection of the values thus obtained, which are presented in Table 3 and Figure 3, reveals that pragmatically speaking, none of the cis-trans energy splitting significantly differs from zero; all values listed in Table 3 are definitely smaller than the “chemical accuracy” of ∼1 kcal/mol. Consequently, it is reasonable to assume that if present, cis and trans conformers of HC_*n*_H^−^ anion chains coexist in extraterrestrial environments.

**Figure 4:**
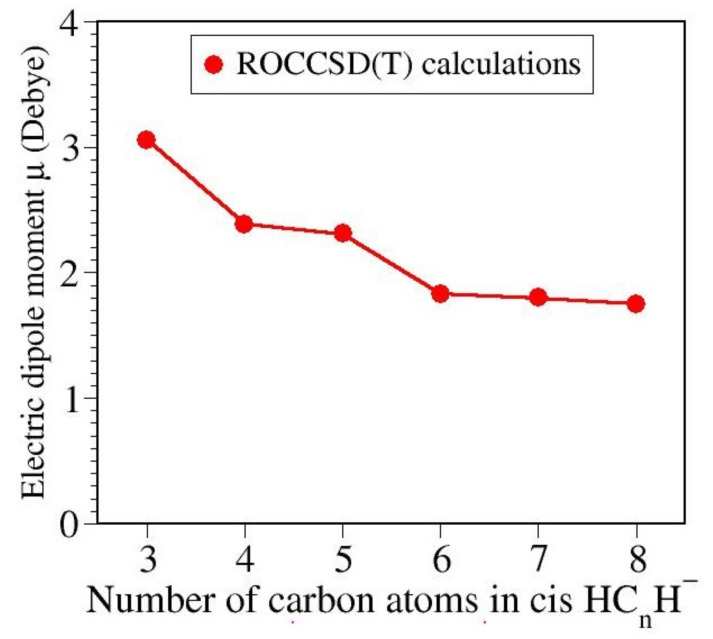
Cis HC_*n*_H^−^ anions with n≤8 possess reasonably large permanent dipole moments, and this can make them observable via rovibrational spectroscopy.

Putting it better, one can rephrase as follows: cis HC_*n*_H^−^ anion conformers can be present in the interstellar medium even if they are slightly higher in energy than their trans counterparts. We said “better” because from the present standpoint, cis anions have a paramount advantage. While the (nearly) centrosymmetric trans anions have (nearly) zero dipole moments, dipole moments of cis HC_*n*_H^−^ anions are substantial; see Table 4 and Figure 4. Above, we wrote “nearly” because the (inherently finite) numerical accuracy prevents us to say whether—in contrast with the well-resolved C${}_{2v}$ symmetry of the cis anions—the trans anions are strictly C${}_{2h}$ symmetric or only approximately.

**Figure A2:**
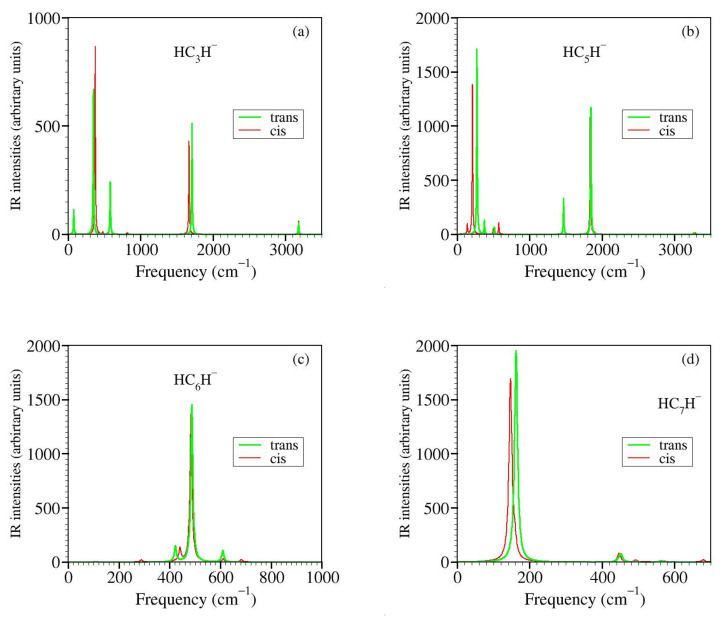
Differences between infrared spectra of cis and trans HC_*n*_H^−^ anion conformers visible here make it possible to experimentally differentiate between them. Curves obtained by using a Lorentzian convolution of halfwidth of 5 ${cm}^{-1}$. (**a**) ${\mathrm{HC}}_{3}H^{-}$; (**b**) ${\mathrm{HC}}_{5}H^{-}$; (**c**) ${\mathrm{HC}}_{6}H^{-}$; (**d**) ${\mathrm{HC}}_{7}H^{-}$.

We do not want to end this section before mentioning that although not very well separated in energy, cis and trans anion isomers have properties sufficiently different from each other enabling experiments to distinguish between them. As illustration, infrared spectra of cis isomers are depicted along with those of trans isomers in Figure A2 of Appendix C. To understand that choosing above infrared spectra as a specific example was not coincidental, let us note that the detection of HC_4_H in ISO observations of CRL 618 [19] relied on laboratory information on the bending mode $\nu_{8}=627.89423\left(10\right)\;{\mathrm{cm}}^{-1}$ [56].

Parenthetically, the difference between the aforementioned value of $\nu_{8}$ with so many digits after the comma and its counterpart at the B3LYP/6-311++G(3df,3pd) level of theory computed by us is $\nu_{8}=624.650\;{\mathrm{cm}}^{-1}$, which may give a (non-astro-)chemist who is not up with astrophysical ways a flavor that only a perfect match between laboratory spectra and observed lines can give a reliable astronomical identification.

## 4. Conclusions

Contrary to previous literature reports [23], we demonstrated that stable HC_*n*_H^−^ anion chains with astrochemically sizes (n≤8) not too large to be accessible via chemical synthesis in extraterrestrial environments are nonlinear. They can be astronomically observed via radio astronomy because they possess sufficiently large permanent dipoles (cf. Table 4) and electron detachment energies (cf. Table 2).

We do hope that this finding will stimulate laboratory experiments aiming at the accurate characterization of HC_*n*_H^−^ anions as a necessary prerequisite for the proper assignment of extraterrestrial signals associated with rovibrational lines. Because any calculation, even obtained with the most sophisticated quantum chemical methods, is unable to give a rovibrational spectrum precise enough to generate a detection in space, it can only help (though it is a lot!) with the laboratory interpretation of an experimental spectrum, which, then, can be used for astronomical observations.

## Figures and Tables

**Table 1:** Enthalpies of formation of the HC_*n*_H and HC_*n*_N chain families computed using the G4 composite model. All values are in kJ/mol.

Number of Carbon Atoms	HC_*n*_H	HC_*n*_N
1	128.668	389.195
2	479.383	228.807
3	370.720	544.779
4	659.255	457.818
5	597.481	729.829
6	863.361	679.914
7	822.640	938.613
8	1080.580	905.162
9	1043.330	1153.130
10	1298.220	1124.610
11	1266.560	1375.680
12	1525.320	1347.420

**Table 2:** Adiabatic electron attachment energies EA computed using the G4 composite model and via ROCCSD(T) at the B3LYP/6-311++G(3df,3pd) minima. Values in eV. Notice that except for HC_4_H^−^, all the other HC_*n*_H^−^ listed possess positive EAs and are therefore stable against electron detachment.

Molecule	G4	ROCCSD(T)
HC_3_H	1.185	1.047
HC_4_H	−0.355	−0.736
HC_5_H ^1^	1.531	1.420
HC_6_H	0.298	0.195
HC_7_H ^2^	1.935	2.029
HC_8_H	0.805	0.667

^1^ Ref. [54] reported EA = 1.51 eV at the CCSD(T)/aug-cc-pVDZ//B3LYP/6-31G level of theory. ^2^ Ref. [55] reported EA = 1.86 eV at the CCSD(T)/aug-cc-pVDZ//B3LYP/6-31G level of theory.

**Table 3:** Cis-trans anion energy splitting $\Delta_{cis-trans}$ estimated within the G4 composite model. Because all these values are smaller than the “chemical accuracy” of ∼1 kcal/mol, one can expect that cis and trans HC_*n*_H anions coexist in space.

Anion	kcal/mol	meV
HC_3_H^−^	0.505	21.9
HC_4_H^−^	0.178	7.7
HC_5_H^−^	−0.260	−11.3
HC_6_H^−^	0.188	8.2
HC_7_H^−^	0.494	21.4
HC_8_H^−^	−0.217	−9.4
HC_9_H^−^	−0.668	29.0

**Table 4:** Dipole moment μ of anion’s cis isomers computed via single-point ROCCSD(T) calculations at the geometry optimized via UB3LYP/6-311++G(3df,3pd).

Cis Anion	Dipole Moment (Debye)
HC_3_H^−^	3.061
HC_4_H^−^	2.385
HC_5_H^−^	2.313
HC_6_H^−^	2.310
HC_7_H^−^	1.801 ^1^
HC_8_H^−^	1.752

^1^ Ref. [55] reported *µ* = 1.63 D at the B3LYP/aug-cc-pVDZ//B3LYP/6-31G level of theory.

**Figure A1:**
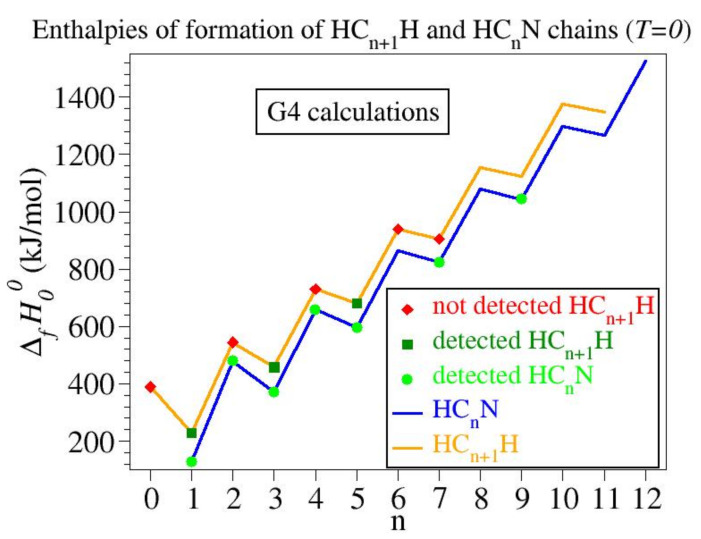
Enthalpies of formation $\Delta_{f}H_{0}^{0}$ presented in Figure 1 are redrawn here to allow direct comparison between the isoelectronic chains HC_*n*+1_H and HC_*n*_N.

## Data Availability

The data that support the findings of this study are available from the author upon reasonable request.

## Appendix A

**Table A1:** Enthalpies of formation of the isoelectronic HC_*n*+_H and HC_*n*_N chain species computed using the G4 composite model. All values are in kJ/mol.

n	HC_*n*+1_H	HC_*n*_N
0	128.668	
1	479.383	389.195
2	370.720	228.807
3	659.255	544.779
4	597.481	457.818
5	863.361	729.829
6	822.640	679.914
7	1080.580	938.613
8	1043.330	905.162
9	1298.220	1153.130
10	1266.560	1124.610
11	1525.320	1375.680
12		1347.420

## Appendix B

Except for HC_4_H^−^—which is less interesting in view of its instability against electron detachment (EA<0, cf. Table 2)—we report below the Cartesian coordinates of the presently considered cis and trans anions; see Table A2, Table A3, Table A4, Table A5 and Table A6.

For open shell optimization, we carried out unrestricted calculations UB3LYP because, according to our experience [26], spin contamination has a negligible impact on the DFT estimates. This obviates the need for restricted open shell ROB3LYP calculations. This sharply contrasts the coupled-cluster (CC) estimates, for which we employed restricted open shell ROCCSD(T) methods; similar to previous studies of related species [26], the impact of spin contamination turned out again to be important, which is a fact that makes less computationally demanding unrestricted UCCSD(T) methods inadequate.

**Table A2:** Cartesian coordinates in Å of the cis and trans HC_3_H^−^ anion conformers optimized at the UB3LYP/6-311++G(3df,3pd) level of theory. Subscripts “before” and “after” label the values of the total spin before and after annihilation of the first spin contaminant.

HC_3_H^−^		cis			Trans	
${\langle S^{2}\rangle }_{\mathrm{before}}$		0.7692			0.7692	
${\langle S^{2}\rangle }_{\mathrm{after}}$		0.7502			0.7502	
**Atom**	**X**	**Y**	**Z**	**X**	**Y**	**Z**
H	0.000000	0.566638	−2.029360	0.000000	−0.431664	−2.096631
C	0.000000	−0.226564	−1.293825	0.000000	0.267660	−1.271822
C	0.000000	−0.080148	−0.000000	0.000000	−0.000000	0.000000
C	0.000000	−0.226564	1.293825	0.000000	−0.267660	1.271822
H	0.000000	0.566638	2.029360	0.000000	0.431664	2.096631

**Table A3:** Cartesian coordinates in Å of the cis and trans HC_5_H^−^ anion conformers optimized at the UB3LYP/6-311++G(3df,3pd) level of theory. Subscripts “before” and “after” label the values of the total spin before and after annihilation of the first spin contaminant.

HC_5_H^−^		cis			Trans	
${\langle S^{2}\rangle }_{\mathrm{before}}$		0.7791			0.7794	
${\langle S^{2}\rangle }_{\mathrm{after}}$		0.7505			0.7505	
**Atom**	**X**	**Y**	**Z**	**X**	**Y**	**Z**
H	0.000000	0.325138	−3.463683	0.000000	−0.389640	−3.424168
C	0.000000	−0.217856	−2.538309	0.000000	0.241108	−2.553977
C	0.000000	0.034756	−1.295056	0.000000	0.047397	−1.297268
C	0.000000	0.154378	0.000000	0.000000	−0.000000	−0.000000
C	0.000000	0.034756	1.295056	0.000000	−0.047397	1.297268
C	0.000000	−0.217856	2.538309	0.000000	−0.241108	2.553977
H	0.000000	0.325138	3.463683	0.000000	0.389640	3.424168

**Table A4:** Cartesian coordinates in Å of the cis and trans HC_6_H^−^ anion conformers optimized at the UB3LYP/6-311++G(3df,3pd) level of theory. Subscripts “before” and “after” label the values of the total spin before and after annihilation of the first spin contaminant.

HC_6_H^−^		cis			Trans	
${\langle S^{2}\rangle }_{\mathrm{before}}$		0.7660			0.7660	
${\langle S^{2}\rangle }_{\mathrm{after}}$		0.7502			0.7501	
**Atom**	**X**	**Y**	**Z**	**X**	**Y**	**Z**
H	0.000000	0.509362	−4.068471	0.000000	−0.429635	−4.080898
C	0.000000	−0.122855	−3.197356	0.000000	0.179751	−3.193660
C	0.000000	0.007350	−1.948075	0.000000	0.017416	−1.948151
C	0.000000	−0.019817	−0.627139	0.000000	0.011855	−0.626980
C	0.000000	−0.019817	0.627139	0.000000	−0.011855	0.626980
C	0.000000	0.007350	1.948075	0.000000	−0.017416	1.948151
C	0.000000	−0.122855	3.197356	0.000000	−0.179751	3.193660
H	0.000000	0.509362	4.068471	0.000000	0.429635	4.080898

**Table A5:** Cartesian coordinates in Å of the cis and trans HC_7_H^−^ anion conformers optimized at the UB3LYP/6-311++G(3df,3pd) level of theory. Subscripts “before” and “after” label the values of the total spin before and after annihilation of the first spin contaminant.

HC_7_H^−^		cis			Trans	
${\langle S^{2}\rangle }_{\mathrm{before}}$		0.7870			0.7871	
${\langle S^{2}\rangle }_{\mathrm{after}}$		0.7509			0.7509	
**Atom**	**X**	**Y**	**Z**	**X**	**Y**	**Z**
H	0.431660	0.000000	−0.921860	0.000000	−0.330843	−4.787382
C	−0.017204	0.000000	0.047244	0.000000	0.144397	−3.832244
C	0.160726	0.000000	1.283517	0.000000	0.020735	−2.589456
C	0.226367	0.000000	2.596912	0.000000	0.016697	−1.275568
C	0.291294	0.000000	3.870346	0.000000	−0.000000	−0.000000
C	0.367671	0.000000	5.146221	0.000000	−0.016697	1.275568
C	0.445956	0.000000	6.455698	0.000000	−0.020735	2.589456
C	0.395178	0.000000	7.708239	0.000000	−0.144397	3.832244
H	0.997605	0.000000	8.593355	0.000000	0.330843	4.787382

**Table A6:** Cartesian coordinates in Å of the cis and trans HC_8_H^−^ anion conformers optimized at the UB3LYP/6-311++G(3df,3pd) level of theory. Subscripts “before” and “after” label the values of the total spin before and after annihilation of the first spin contaminant.

HC_8_H^−^		cis			trans	
${\langle S^{2}\rangle }_{\mathrm{before}}$		0.7695			0.7695	
${\langle S^{2}\rangle }_{\mathrm{after}}$		0.7502			0.7502	
**Atom**	**X**	**Y**	**Z**	**X**	**Y**	**Z**
H	0.000000	0.395448	−5.421548	−0.570041	0.000000	−0.903775
C	0.000000	−0.095135	−4.472275	0.000000	0.000000	0.000000
C	0.000000	0.012485	−3.233941	0.000000	0.000000	1.242922
C	0.000000	−0.007051	−1.908770	0.134072	0.000000	2.561466
C	0.000000	−0.010887	−0.656694	0.247551	0.000000	3.808382
C	0.000000	−0.010887	0.656694	0.364166	0.000000	5.116584
C	0.000000	−0.007051	1.908770	0.477517	0.000000	6.363543
C	0.000000	0.012485	3.233941	0.611671	0.000000	7.681980
C	0.000000	−0.095135	4.472275	0.611324	0.000000	8.925066
H	0.000000	0.395448	5.421548	1.183595	0.000000	9.827551
